# Evaluation of Sit-to-Stand Movement in Older Adults with Locomotive Syndrome Using the Nintendo Wii Balance Board

**DOI:** 10.3390/s23073368

**Published:** 2023-03-23

**Authors:** Go Yamako, Niroshan G. Punchihewa, Hideki Arakawa, Takuya Tajima, Etsuo Chosa

**Affiliations:** 1 Department of Mechanical Engineering, Faculty of Engineering, University of Miyazaki, Miyazaki 889-2192, Japan; 2 Department of Materials and Informatics, Interdisciplinary Graduate School of Agriculture and Engineering, University of Miyazaki, Miyazaki 889-2192, Japan; 3 Department of Rehabilitation, Faculty of Medicine, University of Miyazaki, Miyazaki 889-2192, Japan; 4 Department of Medicine of Sensory and Motor Organs, Division of Orthopedic Surgery, Faculty of Medicine, University of Miyazaki, Miyazaki 889-2192, Japan

**Keywords:** sit-to-stand, force plate, fall prevention, dynamic balance, postural control, locomotive syndrome, falls, center of pressure

## Abstract

Standing up from a chair is a mechanically demanding daily motion, and its biomechanics represent motor performance. In older adults with locomotive syndrome (LS), sit-to-stand (STS) movement with adequate postural control is essential to prevent falls. This study evaluated the characteristics of dynamic balance during STS movement on older adults with LS. A total of 116 participants aged ≥65 years were divided into Non-LS, LS stage 1, and LS stage 2 groups using the LS risk test. The participants were instructed to stand on the Nintendo Wii Balance Board as quickly as possible, and the STS movement was quantified using the vertical ground reaction force (VGRF) and center of pressure (CoP). The STS score, which represented dynamic balance, was significantly different among the groups (*p* < 0.001). The rate of VGRF development was significantly lower in the LS stages 1 and 2 than in the Non-LS group (*p* < 0.001). On the other hand, the total distance of the CoP path did not differ among the groups (*p* = 0.211). These findings indicated a reduction of postural control in older adults with LS. The STS score emphasized the importance of balance training to prevent falls in older adults with LS.

## 1. Introduction

Aging is characterized by biological changes, such as reductions in muscle mass and strength and the loss of postural control and mobility [1,2]. The functional decline of the locomotor organs leads to difficulties in performing activities of daily living (ADL) and, consequently, decreased quality of life. In 2007, Japan was reported to have an aging rate that exceeded 22% and faced a super-aged society [3]. Therefore, the Japanese Orthopaedic Association (JOA) proposed the concept of locomotive syndrome (LS) to define individuals at risk of requiring healthcare support or nursing care services because of difficulties in living independently as a consequence of problems in locomotive organs, such as the muscles, bones, joints, and nerves [3]. A previous study estimated that a total of 47 million people aged 40 years and older were candidates for developing LS in Japan [4]. The JOA developed a risk test to diagnose the progression of LS (i.e., Non-LS, LS stage 1, or LS stage 2) [5,6]. LS stages 1 and 2 represent the beginning of and progressive decline in mobility, respectively. The prevalence rate of LS stages 1 and 2 in community-dwelling older adults was estimated at 69.8% and 25.1%, respectively [7]. Older adults with LS may have reduced postural control to safely perform ADL, and this subsequently results in falls.

Postural control is a complex process based on the dynamic interaction of the sensory (i.e., visual information, vestibular system, and somatosensory system) and motor (i.e., neuromusculoskeletal) systems [8]; it is the act of maintaining, achieving, or restoring a state of postural or dynamic balance [9]. Deterioration or impairment of the postural control system weakens the ability to maintain balance and increases the risk for falls and fall-related injuries. Almost 30% of people aged ≥65 years fall at least once per year [10,11], and approximately 20% of the falls were severe, including serious injuries and fractures, leading to immobility or death [10,12]. Therefore, evaluating the postural control of older adults is important to prevent the progression of LS.

Among the ADL, standing up from a chair is vital; a decreased ability to perform this movement is associated with falls [10], hip fracture [13], hospitalization, or even death in older adults [14]. Moreover, the sit-to-stand (STS) movement has been identified as mechanically demanding, because it requires significant muscle strength, a wide joint motion, and dynamic balance [15,16]. Accordingly, an age-related decrease in muscle strength and balance control have frequently been associated with difficulties in completing STS movements [17,18,19]. Older people change their strategies for the STS movement [20,21,22]. In general, they stand up with greater flexion of the trunk, aiming to minimize the demands on the muscle strengths. Hughes et al. reported that the functionally impaired elderly increase their momentum in rising by increasing their hip flexion; they simultaneously attempt to increase their stability by taking more time to rise and shortening the distance between their center of mass and base of support at lift-off [22]. Therefore, the STS movement has been identified as a valuable source of an individual’s postural control [20,23,24,25].

We previously developed a quantitative method to estimate dynamic balance during the STS movement based on the vertical ground reaction force (VGRF) and center of pressure (CoP) that were measured by the Nintendo Wii Balance Board (WBB; Nintendo, Kyoto, Japan) [26]. To evaluate dynamic balance, we proposed the new index STS score, which is defined by a combination of the rate of the VGRF development (RFD) and the CoP path length during STS movement. A previous study on 503 healthy individuals aged 20–88 years revealed an age-related decline in the STS score [26]. However, to our best knowledge, there had been no study to clarify the difference in the STS score between individuals with and without LS. The purpose of the present study was to investigate the characteristics of the STS movement in older adults with LS. In addition, we conducted conventional functional tests, including the timed up and go (TUG) test, the one-leg standing (OLS) test with eyes open, and the handgrip strength test. We hypothesized that these functional motor variables would differ according to the severity of LS.

## 2. Materials and Methods

### 2.1. Participants

The participants were recruited from nine senior clubs. The clubs were community-based social groups that were financially supported by local governments. The exclusion criteria were age <65 years and the presence of any known musculoskeletal or neuromuscular conditions that would limit mobility or the ability to perform the STS movement. The research protocol of this study followed the Declaration of Helsinki and was approved by the ethics committee of our university (reference number: 2014–231). All participants provided written informed consent. The participants performed the tests at random. They were allowed to take a break between each test to perform the measurements in a safe manner. Of the 163 individuals recruited, 11 were excluded according to the exclusion criteria. Then, we enrolled 116 out of 152 participants by a simple random sampling without gender gap (resulting in 58 participants for each gender).

### 2.2. The Locomotive Syndrome Risk Test

The participants were categorized into three groups according to the status of LS, as determined by the LS risk test [5,27], which comprised the following three parts: (1) a 25-item geriatric locomotive function scale (GLFS-25), (2) the two-step test, and (3) the stand-up test. Summaries of the three tests are provided below.

1. GLFS-25: The GLFS-25 is a self-administered comprehensive measure that comprised 25 items, including 4 questions on pain, 16 questions on ADLs, 3 questions on social functions, and 2 questions on mental health status. Each question was graded with a five-point scale, from no impairment (0 points) to severe impairment (4 points). The points for each question were added to obtain the total score (minimum = 0; maximum = 100). Therefore, a higher score reflected decreased mobility.
2. Two-step test: The two-step test measured the stride length to assess walking ability, including the muscle strength, balance, and flexibility of the lower limbs. The participants were instructed to take two steps as long as possible and then to align both feet. The score was calculated by dividing the maximum length of the two steps by the participant’s height.
3. Stand-up test: The stand-up test assessed leg strength. From four different seated positions (chair height of 40, 30, 20, and 10 cm), the participants were asked to stand on one leg and then on both legs for each chair height. Lowering the chair height led to a greater biomechanical demand while standing up from a seated position. The participants were instructed to avoid leaning back while maintaining a standing posture for three seconds. They were scored based on their height level using one leg and both legs. The scores ranged from 0 to 8, depending on their difficulty in standing up [28]. Higher stand-up scores indicate better leg strength. In this study, the stand-up score ranged from 1 to 6 points.

After these tests, the participants were diagnosed with their LS severity based on the JOA protocol [6]. LS stage 1 indicated the beginning of a decline in mobility and was assigned if the participant could not perform the stand-up test on one leg from a 40-cm-high chair, if the GLFS-25 score was ≥7, or if the two-step test score was <1.3. LS stage 2 represented progressive decline in mobility and was assigned if the participant could not perform the stand-up test on both legs from a 20-cm-high chair, if the GLFS-25 score was ≥16, or if the two-step test score was <1.1.

### 2.3. Sit-to-Stand Movement Test

The participants were instructed to stand up from a chair as quickly as possible, immediately recover their balance, and stand as still as possible in an upright posture with their arms crossed over their chest for five seconds. They were seated on an armless, backless chair with both feet placed shoulder-width apart on the WBB in 20° dorsiflexion. The seat height was adjusted according to the participant’s knee height by using 2-cm-thick wooden boards placed under WBB or the chair.

The WBB, which was originally designed as a video game controller, has been increasingly used to assess postural control in rehabilitation [29,30,31,32,33,34]. It comprises a rigid platform with four strain gauge-based vertical load transducers located on each corner. For this study, we used the WBB to calculate the VGRF and the CoP during the STS movement. WBB data were streamed to a laptop at approximately 100 Hz. Before data recording, participants were allowed to practice the procedure. Each participant performed two trials with a one-minute interval.

**Figure 1:**
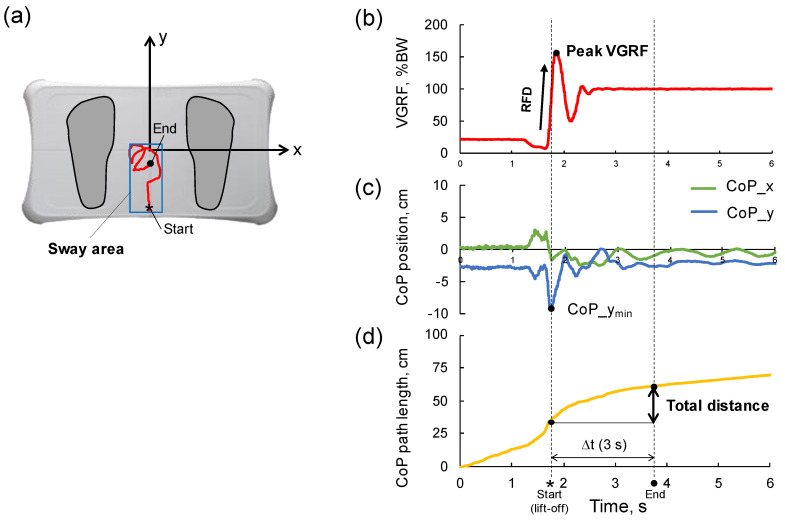
Typical graphs and definitions of the sit-to-stand movement. (**a**) The CoP path is shown with the coordinate system of the Wii Balance Board. The x and y axis are medio-lateral and antero-posterior direction, respectively. The CoP moves from posterior (* indicates start point) to anterior (● indicates end point). The end point (●) indicates the CoP at 3 s after lift-off. The rectangle in blue indicates the CoP sway area (sway area). (**b**) The vertical ground reaction force (VGRF) is normalized by the body weight (%BW). The rate of VGRF (RFD) is defined as an initial slope of the curve. (**c**) The CoP position in the medio-lateral (CoP_x) and antero-posterior (CoP_y) directions are plotted. The CoP_y_min_ is the minimum value of the CoP_y, which indicates a time of lift-off from a chair. (**d**) The CoP path length increases with time. The total distance was calculated as the CoP path length during Δ*t*.

To estimate postural control, the STS movement was quantified by parameters which were calculated using the CoP path (Figure 1a), VGRF (Figure 1b), CoP position in x (CoP_x) and y (CoP_y) directions (Figure 1c), and the CoP path length (Figure 1d). The CoP path length was defined as the sum of the distances between consecutive points on the CoP path. The VGRF was normalized to the participant’s body weight. For this work, due to ease of determination it was assumed that the beginning of the motion (lift-off from the chair) coincided with the CoP_y component having its minimum value (see Figure 1c) (1).

(1) $$t_{\mathrm{lift}-\mathrm{off}}=t\left(\mathrm{CoP}\_y_{\min}\right)$$

The time interval for the measurements of the CoP related variables was set from a time of lift-off to + 3 s (Figure 1) (2).

(2) $$∆t=\left[t_{\mathrm{lift}-\mathrm{off}},\;t_{\mathrm{lift}-\mathrm{off}}+3\right]\;\left(s\right)$$

#### 2.3.1. Rate of Vertical Ground Force Development

The RFD (BW/s) was defined as the linear slope of the VGRF–time curve (Figure 1b). In this study, the linear part of the curve was set as the time period between 25% of the peak VGRF ($t_{25\%{\mathrm{VGRF}}_{\mathrm{peak}}}$) and 90% of the peak VGRF ($t_{90\%{\mathrm{VGRF}}_{\mathrm{peak}}}$) (3). The peak VGRF was also used as a parameter to evaluate postural control.

(3) $$RFD=\frac{0.65\times peak\;VGRF}{t_{90\%{\mathrm{VGRF}}_{\mathrm{peak}}}-t_{25\%{\mathrm{VGRF}}_{\mathrm{peak}}}}\left(BW/s\right)$$

#### 2.3.2. Total Distance

The total distance (cm) during STS movement was defined as the CoP path length (cm) during Δ*t* (4). At 3 s from the time of lift-off ($t_{\mathrm{lift}-\mathrm{off}}$), the CoP path length was substantially stable (not a plateau), which indicates that the upright posture of the participants was stabilized (Figure 1c).

(4) $$Total\;distance\;=\;CoP\;path\;length\left(∆t\right)\;\left(\mathrm{cm}\right)$$

The total distance_x and _y defined as the CoP pass length for the *x*-axis (mediolateral direction) and the *y*-axis (anteroposterior direction) during Δ*t*.

#### 2.3.3. Sway Area

The sway area (cm^2^) was defined as a rectangle surrounded the CoP sway during STS movement (Figure 1a). This parameter was calculated by the product of the range of CoP_x and CoP_y during Δ*t*. During the STS motion, the CoP moved from posterior to anterior and stayed with a slight sway at upright posture.

#### 2.3.4. Sit-to-Stand Score

The STS score (BW/(ms)) comprised the RFD and the total distance to quantify STS movement (5).

(5) $$STS\;score\;=\;\frac{RFD}{Total\;distance}\;\left(\mathrm{BW}/\mathrm{ms}\right)$$

Higher STS scores indicate better dynamic balance. In general, these two indices have a tradeoff relationship. During STS movement, the horizontal momentum increases with an increase in movement speed (i.e., RFD). This induces a difficulty in controlling posture and minimizing sway (i.e., total distance) [21,35].

The variables were calculated from each set of trial data using a custom MATLAB program (MathWorks, Natick, RI, USA). Participants were allowed two trials, and the maximum STS score was used as representative data to analyze the best performance of the STS movement.

### 2.4. Timed Up and Go Test

The TUG test is a well-known clinical test that was developed to improve the evaluation of functional performance and mobility [36]. This test measures the time needed to rise from a chair, walk 3 m, turn around, walk back, turn around, and sit down again. A shorter TUG time indicates better performance. The study participants were instructed to walk as quickly and safely as possible. For each participant, a practice trial was followed by two timed trials, and the fastest trial was selected for analysis.

### 2.5. One-Leg Standing Test with Eyes Open

The OLS test measures the time while standing on one lower limb without extra support. This test is a widely-used clinical tool to easily quantify postural control in a static position [37]. In this study, the OLS time with eyes open was measured twice for the dominant leg up to a maximum of 120 s; the longer time was used for analysis.

### 2.6. Handgrip Strength Test

The handgrip strength indicates motor function and ADL performance [38] and has been reported to significantly correlate with leg muscle strength in the elderly [39]. In this study, strength was measured bilaterally in a standing position using a handgrip dynamometer. Handgrip strength was assessed by conducting two trials for each hand, and the maximum value obtained across both trials was considered to represent the individual’s overall handgrip strength.

### 2.7. Statistical Analyses

In this study, we examined the difference in each variable among the Non-LS, LS stage 1, and LS stage 2 groups. Thereafter, we evaluated the correlation among variables. All data were presented as mean ± standard deviation. The Shapiro–Wilk test was performed to confirm normal distribution of the data. When the data showed a normal distribution, one-way analysis of variance, followed by Fisher’s least significant difference test, was conducted to compare the groups. Otherwise, a Kruskal–Wallis test followed by a multiple comparison test with Bonferroni correction was performed. Post hoc statistical powers were calculated. Considering the effect size, which was determined from the results, and with *α* = 0.05, powers of >0.95 were obtained for variables that were significantly different among the groups (RFD, peak VGRF, STS score, TUG time, OLS time, and handgrip strength).

Spearman’s rank correlation coefficient was used to analyze the relationships among variables. The difference in sex ratio among the groups was examined using the chi-square test.

Statistical analyses were performed using the Statistical Package for the Social Sciences version 28.0 (IBM SPSS, Chicago, IL, USA). The statistical significance was set as *α* = 0.05.

## 3. Results

### 3.1. Baseline Characteristics of the Enrolled Participants

The 116 participants had a mean age of 75.9 ± 5.8 years (range, 65–88 years) and a mean body mass index of 23.8 ± 3.1 kg/m^2^. A total of 91 participants (78.4%) were diagnosed as LS. The condition severity was Non-LS in 21.5%, LS stage 1 in 40.5%, and LS stage 2 in 37.9%. The characteristics and results of the LS risk test are summarized in Table 1. There was a significant difference in the sex ratio among the LS groups (*p* = 0.006).

### 3.2. Sit-to-Stand Movement and Parameters

At the start of the STS movement, the VGRF decreased with hip flexion. Thereafter, hip and knee joint extensions started, and the VGRF reached a peak value within half a second and oscillated around the body weight. Within three seconds from the start of the STS movement, the oscillation stabilized and the CoP path length became substantially stable, although not a plateau. Similar waveforms were observed among groups; however, differences were noted in the STS parameters (Table 2). The RFD, peak VGRF, and STS score differed significantly among the LS groups (*p* < 0.001 for all). The RFD was significantly lower in the LS stage 2 group than in the Non-LS (*p* = 0.000) and LS stage 1 (*p* = 0.017) groups. The peak VGRF and STS score were significantly higher in the Non-LS group than in the LS stage 1 (*p* = 0.028 for both) and LS stage 2 (*p* = 0.000 and *p* = 0.008, respectively) groups. However, there were no significant differences in the total distance, total distance_x, total distance_y, and sway area of the *CoP* among the groups.

### 3.3. Results of the Conventional Functional Tests

The TUG time differed significantly among the LS groups (*p* < 0.001, Table 3). It was significantly shorter in the Non-LS group than in the LS stage 1 (*p* < 0.001) and LS stage 2 (*p* < 0.001) groups. It was significantly shorter in the LS stage 1 group than in the LS stage 2 group (*p* < 0.001).

The OLS time differed significantly among the LS groups (*p* < 0.001, Table 3). It was significantly longer in the Non-LS group than in the LS stage 1 (*p* = 0.001) and LS stage 2 (*p* < 0.000) groups. It was significantly longer in the LS stage 1 group than in the LS stage 2 group (*p* = 0.008).

The handgrip strength differed among the LS groups (*p* = 0.021, Table 3). It was significantly greater in the Non-LS group than in the LS stage 1 (*p* < 0.001) and LS stage 2 (*p* < 0.001) groups. It was significantly greater in the LS stage 1 group than in the LS stage 2 group (*p* < 0.001).

### 3.4. Correlation of the Sit-to-Stand Score with the Conventional Functional Tests

**Figure 2:**
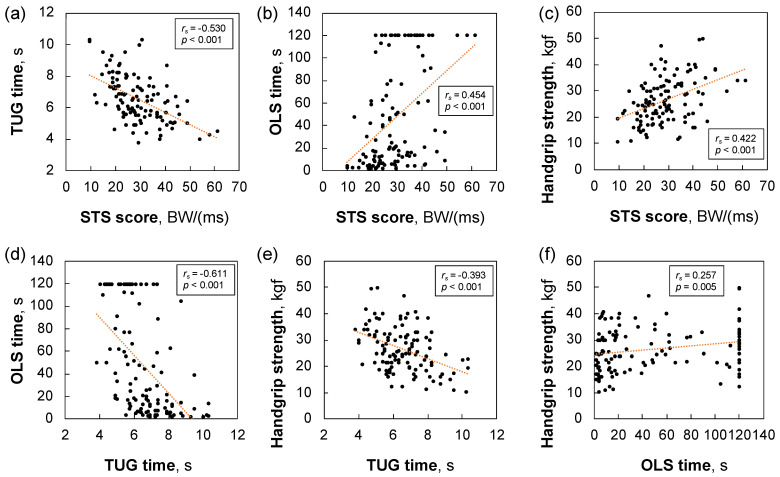
Scatter plots of the variables used for Spearman’s rank correlation coefficient between (**a**) STS score and TUG time, (**b**) STS score and OLS time, (**c**) STS score and handgrip strength, (**d**) TUG time and OLS time, (**e**) TUG time and handgrip strength, and (**f**) OLS time and handgrip strength. STS, sit-to-stand; TUG, timed up and go; OLS, one-leg standing.

The Spearman’s rank correlation coefficients between variables are shown in Table 4. The STS score was significantly associated with the TUG time (*r_s_* = −0.530, *p* < 0.001), OLS time (*r_s_* = 0.454, *p* < 0.001), and handgrip strength (*r_s_* = 0.422, *p* < 0.001) (Figure 2).

## 4. Discussion

LS is a condition that places an individual at risk of needing healthcare support because of problems with the neuromusculoskeletal system [3]. In the present study, we assessed the postural control of elderly individuals with LS by quantifying their STS movement. Our results showed that the STS score, which represents dynamic balance, was significantly lower in the participants who had LS than in those who had no LS. This finding indicates that older adults with LS has relatively poor postural control, because they could not stand up from a chair quickly and stay still. Therefore, older adults with LS would need balance training or other forms of training to prevent falls. Our results add support to the fact that the incidence of falls in the older population was reported to be higher in those with LS than in those without LS [40]. In older adults, physical intervention using general balance exercises can decrease the incidence of falls [41], while patient motivation and adherence are important factors for improvement. Therefore, to improve and maintain balance in older adults with LS, fun and dynamic CoP control-based exercise games (exergames) or exercise robots (exerbots) may be effective in preventing falls [42,43].

The ground reaction force in STS movement is associated with lower extremity strength and balance function [44,45]. The RFD, which is an index of the capacity for rapid muscle force production and was defined as a slope of the VGRF–time curve in this study, differed significantly among the LS groups. This indicated that the STS movements of older adults were slower in those with LS than in those without LS. The finding is consistent with the results of a recent kinematic study on the TUG test in LS cases by Kataoka et al. [46]. They reported that the angular velocity of the hip and knee extension during STS phase in the LS stage 1 group was significantly lower than in the Non-LS group. In addition, we found that the peak VGRF significantly differed among the LS groups. This parameter represents the maximal force that pushes the body upwards when standing from a chair. It reflects the strength and power in the lower limbs [44] and can predict the risk for fall [47]. Therefore, our findings implied that older adults with LS have relatively weak muscle strength.

CoP measures have been used as the indicators of balance control [48,49,50]. Degani et al. concluded that CoP displacement was able to capture subtle effects of the natural process of aging on the mechanisms of postural control [51]. The physically inactive older women exhibited greater sway velocity compared with the young women [52]. Tanaka et al. reported that an increase in back-and-forth sway was an important factor for LS risk [53]. Moreover, our previous study revealed an age-related decrease in the total distance during the STS movement [26]. Thus, we expected differences in CoP measures between older adults with LS and without LS. However, there were no significant differences in the total distance and the sway area among the groups. These results suggested that older adults with LS decreased their movement speed to maintain postural stability and accomplish the STS movement safely. This strategy is confirmed by a previous study by Shultz et al. [54]. They demonstrated that during slow chair rises elderly subjects use a strategy that enhances stability, by locating the center of pressure more anteriorly at lift-off. Therefore, using the CoP parameters during the STS movement alone may misrepresent an individual’s postural control. The results emphasize the importance of using the STS score, which is a combination of the VGRF and the CoP parameters.

The relationship between simple physical functional tests and the STS score in older adults was also examined. Consistent with previous studies [55,56,57,58,59], this study showed significant differences in the TUG time, OLS time, and handgrip strength among the groups. A clinical prediction rule using these physical tests to assess the severity of LS in older adults was previously proposed [56]. Our results showed significant correlations between the STS score and these physical measures. Therefore, the STS score may be a candidate tool to evaluate and monitor the severity of LS in older adults.

Several limitations of this study should be addressed. Participants were recruited from senior clubs. These individuals may have been health conscious and may have had better motor abilities, compared with the general population, because they joined the club voluntarily. We did not evaluate the size of the feet and the inter-foot distance of each participant. These parameters would influence the results of CoP parameters. The correlations and effects of the CoP parameters should be further evaluated. This study is a cross-sectional study. Thus, the incidence of falls was not evaluated. The relationship between the STS score and falls risk is of great interest. Further studies addressing these issues are needed.

## 5. Conclusions

This study investigated the characteristics of the STS movement in people aged ≥65 years with LS. Our results demonstrated a significant difference in dynamic balance during the STS movement between older adults with and without LS. The RFD was significantly lower in those with LS than in those without LS. On the other hand, the total CoP distance was not different among the severity groups of LS. These findings indicated that older adults with LS needed to decrease their movement speed to maintain balance and accomplish the STS movement safely, thereby implying reduced postural control. The STS score emphasized the importance of balance training to prevent falls in older adults with LS.

## Figures and Tables

**Table 1:** Characteristics of the enrolled participants (*n* = 116) according to the LS severity.

	Non-LS (*n* = 25)	LS Stage 1 (*n* = 47)	LS Stage 2 (*n* = 44)	Total (*n* = 116)
Age, years	71.4 ± 5.0	75.8 ± 5.2	78.7 ± 5.3	75.9 ± 5.8
Sex (men/women)	17/8	27/20	14/30	58/58
Mass, kg	57.0 ± 8.7	58.6 ± 9.8	55.1 ± 9.4	57.0 ± 9.5
Height, cm	158.1 ± 7.8	157.1 ± 8.2	150.6 ± 8.4	154.9 ± 8.8
BMI, kg/m^2^	22.9 ± 3.1	23.6 ± 2.9	24.4 ± 3.2	23.8 ± 3.1
GLFS-25	1.8 ± 1.8	5.7 ± 4.3	21.6 ± 16.3	10.9 ± 13.4
Two-step score	1.47 ± 0.10	1.30 ± 0.12	1.15 ± 0.15	1.28 ± 0.17
Stand-up score	5.3 ± 0.5	3.9 ± 0.8	3.3 ± 1.1	4.0 ± 1.2

LS, locomotive syndrome; BMI, body mass index; GLFS-25, geriatric locomotive function scale.

**Table 2:** STS variables for each LS group.

	Non-LS	LS Stage 1	LS Stage 2	*p*-Value
RFD, BW/s	9.8 ± 2.3	8.6 ± 2.3 *	6.9 ± 1.8 *#	<0.001
Peak VGRF, %BW	135.8 ± 11.5	129.1 ± 11.8 *	124.1 ± 7.9 *	<0.001
Total distance, cm	28.6 ± 6.6	30.9 ± 6.8	29.3 ± 7.6	0.211
Total distance_x, cm	13.6 ± 3.6	14.7 ± 4.1	14.4 ± 4.2	0.461
Total distance_y, cm	22.1 ± 5.7	23.7 ± 5.4	21.9 ± 6.7	0.149
Sway area, cm^2^	38.4 ± 16.8	45.6 ± 30.7	41.6 ± 30.0	0.575
STS score, BW/(ms)	35.6 ± 10.3	28.8 ± 8.8 *	25.0 ± 8.8 *	<0.001

* *p* < 0.05 (vs. Non-LS), # *p* < 0.05 (vs. LS stage 1). LS, locomotive syndrome; RFD, rate of vertical ground reaction force development; BW, body weight; VGRF, vertical ground reaction force; STS, sit-to-stand.

**Table 3:** Conventional functional motor measures for each LS group.

	Non-LS	LS Stage 1	LS Stage 2	*p*-Value
TUG time, s	5.3 ± 0.9	6.4 ± 1.1 *	7.5 ± 1.4 *#	<0.001
OLS time, s	97.0 ± 38.2	42.7 ± 39.8 *	22.2 ± 30.0 *#	<0.001
Handgrip strength, kgf	32.1 ± 8.1	27.8 ± 8.0 *	21.7 ± 6.7 *#	<0.001

* *p* < 0.05 (vs. Non-LS), # *p* < 0.05 (vs. LS stage 1). LS, locomotive syndrome; TUG, timed up and go; OLS, one-leg standing.

**Table 4:** Spearman’s rank correlation coefficients (*r_s_*) among the variables.

	STS Score	TUG Time	OLS Time	Handgrip Strength
STS score	1.000	−0.530 **	0.454 **	0.422 **
TUG time	−0.530 **	1.000	−0.611 **	−0.393 **
OLS time	0.454 **	−0.611 **	1.000	0.257 **
Handgrip strength	0.422 **	−0.393 **	0.257 **	1.000

** *p* < 0.01. STS, sit-to-stand; TUG, timed up and go; OLS, one-leg standing.

## Data Availability

Not applicable.
